# High sensitivity methods to quantify chloroquine and its metabolite in human blood samples using LC–MS/MS

**DOI:** 10.4155/bio-2018-0202

**Published:** 2019-03-15

**Authors:** Karnrawee Kaewkhao, Kesinee Chotivanich, Markus Winterberg, Nicholas PJ Day, Joel Tarning, Daniel Blessborn

**Affiliations:** 1 Mahidol-Oxford Tropical Medicine Research Unit, Faculty of Tropical Medicine, Mahidol University, Bangkok, Thailand; 2 Department of Clinical Tropical Medicine, Faculty of Tropical Medicine, Mahidol University, Bangkok, Thailand; 3 Centre for Tropical Medicine & Global Health, Nuffield Department of Clinical Medicine, University of Oxford, Oxford, UK

**Keywords:** chloroquine, dried blood spots, human blood, LC–MS/MS, malaria, method validation

## Abstract

**Aim:**

Chloroquine is an antimalarial drug used in the treatment of *Plasmodium vivax* malaria. Three methods to quantify chloroquine and its metabolite in blood matrices were developed and validated.

**Methodology & results:**

Different high-throughput extraction techniques were used to recover the drugs from whole blood (50 μl), plasma (100 μl) and dried blood spots (15 μl as punched discs) followed by quantification with LC–MS/MS. The intra- and inter-batch precisions were below 15%, and thus meet regulatory acceptance criteria.

**Conclusion:**

The developed methods demonstrated satisfactory validation performance with high sensitivity and selectivity. The assays used simple and easy to automate extraction techniques. All methods were reliable with robust performance and demonstrated to be suitable to implement into high-throughput routine analysis of clinical pharmacokinetic samples.

Malaria is still a major public health problem worldwide, resulting in an estimated 445,000 annual deaths in 2016 [1,2]. Chloroquine was once the most extensively used antimalarial drug, due to its low cost, high efficacy and relative safety [3]. It was later discontinued for the treatment of *Plasmodium falciparum* infections in most countries worldwide due to increasing drug resistance [3,4]. However, it has been reported that a prolonged absence of chloroquine in endemic areas can lead to a reversal of resistance in the parasite population, providing a renewed potential to treat *P. falciparum* infections [5]. Chloroquine is still the main first-line therapy recommended for the treatment of *Plasmodium vivax* infections [1,3]. However, chloroquine resistance has been reported in *P. vivax* in Brazil [6], Ethiopia [7], Indonesia [8], Malaysia (Borneo) [9], Myanmar [10,11], Thailand [12], Papua New Guinea [13] and Peru [14]. Chloroquine belongs to the 4-aminoquinoline group of antimalarial drugs (Figure 1). The major active metabolite of chloroquine, generated by CYP450 CYP2C8 and CYP3A4/5 enzymes, is desethylchloroquine [15]. Both chloroquine and desethylchloroquine are slowly eliminated, with a terminal elimination half-life of approximately 30–60 days [16]. They are mainly bound to platelets, erythrocytes, thrombocytes and granulocytes, similar to other quinoline antimalarial drugs, resulting in increased concentrations in infected or uninfected blood cells that are about two- to five-times higher than what can be found in plasma [16–18]. Figure 1 shows the molecular structure of chloroquine and desethylchloroquine.

**Figure 1.**
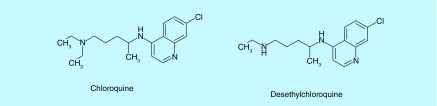
**Molecular structure of chloroquine and desethylchloroquine.**

Several quantification methods of chloroquine in biological matrices have been described previously [17,19–27]. Early publications of bioanalytical methods commonly used extraction procedures, such as protein precipitation and liquid–liquid extraction, which often leave protein residues in the extracted sample [19,20,22,24,25,28]. Liquid–liquid extraction is also labor intensive and time consuming. A separation and detection method consisting of LC coupled with UV or fluorescence detection are easy to operate but provide only low sensitivity and selectivity, and often require large sample volumes to achieve adequate sensitivity for quantification of clinical pharmacokinetic samples [19–25,28]. A recent publication with diode array detector used a more powerful sample extraction technique (e.g., SPE). However, this method still proved less sensitive (LLOQ of 10 ng/ml) using a large injection volume (50 μl) for improved sensitivity [29]. The introduction of mass spectrometric detection has become popular for its high sensitivity and selectivity that is useful for pharmacokinetic studies. Several mass spectrometric methods have been published for chloroquine determination, but those were simultaneous analysis with other antimalarial drugs [30,31]. Simultaneous analysis often leads to compromises, for example, a general extraction method needs to be used that can lead to severe matrix effects [30]. A large injection volume to improve sensitivity and prolonged analysis time, for example, 19–21 min/sample due to added chromatography column washout period, to reduce memory effects or sample carryover, were some of the compromises met [30,31].

Here we present an optimized protocol for the quantification of chloroquine and its metabolite, desethylchloroquine, in plasma, whole blood and dried blood spots (DBS) using LC–MS/MS. Three different extraction techniques were used to ensure high-throughput and optimal recoveries of the drugs from the different biological matrices. The use of MS for the detection of the drug molecules provides higher sensitivity, selectivity, and requires smaller sample volumes. The methods described here were developed and optimized for implementation in high-throughput routine settings and were validated in accordance to the Guidance for Industry, Bioanalytical Method Validation (US FDA, 2001) [32] and the Guidance on Bioanalytical Method Validation (European Medicines Agency, London, UK, 2012) [33].

## Materials & methods

### Chemicals & reagents

Chloroquine and desethylchloroquine were obtained from AlsaChim (Illkirch, France). The stable isotope-labeled internal standards, chloroquine-D4-diphosphate salt and desethylchloroquine-D4, were obtained from Santa Cruz Biotechnology (TX, USA). All solvents and chemicals were of MS grade, except ethyl acetate, which was HPLC grade, and ammonia solution (25%), which was analytical grade. Water, acetonitrile, methanol and ethyl acetate were obtained from JT Baker (NJ, USA). Formic acid (98–100%) and ammonium formate were obtained from Fluka (Sigma-Aldrich, MO, USA). Ammonia solution (25%) was used to prepare ammonium hydroxide 0.5 M (Merck, Darmstadt, Germany). Blank whole blood and plasma were obtained from Thai Red Cross, Bangkok, Thailand with citrate phosphate dextrose as anticoagulant. For other anticoagulants, EDTA, fluoride-oxalate, fluoride-heparin, Na-heparin and Li-heparin were collected from healthy volunteers at the Faculty of Tropical Medicine, Mahidol University, Thailand. Ethical approval for the method development and validation was sought from the Ethics Committee of the Faculty of Tropical Medicine, Mahidol University, Thailand (certificate no. MUTM 2017-014-01 and approval no. TMEC 16–095).

### Equipment

The following SPE columns were used for sample extraction: 100 mg, 1 ml, carboxylic acid bonded sorbent (CBA) fixed 96-wellplate (Biotage, Uppsala, Sweden) for whole blood, Phree Phospholipids Removal 96-wellplate, 8E-S133-TGB (Phenomenex, CA, USA) for DBS and ISOLUTE^®^ SLE^+^ 96-well plate, 820-0200-P01, IST (Biotage, Uppsala, Sweden) for plasma. A Freedom Evo 200 platform liquid handler (TECAN, Mannedorf, Switzerland) was used to automate the sample preparation and extraction. A Robotic Punch Instrument (BSD600-Duet Semi-Automated, Queensland, Australia) was used to obtain samples from the DBS. A TurboVap^®^96 (Biotage) was used to evaporate the eluted sample.

### Preparation of standards, working solutions, calibration standards & quality control samples

Stock solutions (1 mg/ml) of chloroquine, desethylchloroquine and their stable isotope-labeled internal standards were prepared in acetonitrile–water (50–50, v/v) containing 0.5% formic acid and stored at -80°C. Working solutions were prepared from the stock solution using acetonitrile–water (50–50, v/v) as dilution solution and then used for the spiking of whole blood, plasma and whole blood for DBS.

Unless otherwise stated, blank blood from healthy volunteers with EDTA as anticoagulant was used. Plasma was obtained by centrifugation of whole blood at 1500–2000 × *g* for 10 min [34,35]. Whole blood applied on chromatography filter paper Whatman (31 ET Chr, DMPK-C, 903 Protein saver and 3 MM Chr; Whatman, Buckinghamshire, UK) and an alternative brand, Ahlstrom 226 (PerkinElmer, MA, USA) was used for DBS technique. The calibration curves of chloroquine/desethylchloroquine were 2.56–1220/3.36–1220 ng/ml, 1.41–610/1.41–610 ng/ml and 1.82–1552/2.95–1552 ng/ml in whole blood, plasma and DBS, respectively. The final volume of working solution in blank blood was kept below 5% in all samples.

### Extraction procedure

Whole blood, plasma or punched discs of DBS were aliquoted into 96-well plates and processed using an automated liquid handler platform (Freedom Evo 200) as described below.

Whole blood (50 μl) was aliquoted into a 96-wellplate and 100 μl of water containing stable isotope-labeled internal standard (desethylchloroquine-D4 25.8 ng/ml and chloroquine-D4 72.5 ng/ml) was added, followed by 450 μl of ammonium carbonate 20 mM. The plate was mixed on Mixmate (Eppendorf, Hamburg, Germany) at 1000 r.p.m. for 2 min and centrifuged at 1100 × *g* for 2 min (i.e., extraction-ready samples). CBA-fixed SPE 96-wellplate cartridges were conditioned with methanol (1 ml) followed by ammonium carbonate 20 mM (1 ml). Each buffer-diluted whole blood sample (200 μl) was loaded onto the conditioned CBA SPE 96-wellplate and subsequently washed with ammonium carbonate 20 mM (1 ml), ammonium carbonate 20 mM–methanol (20–80, v/v; 1 ml) and methanol–water (50–50, v/v; 1 ml). Full vacuum (10-inch Hg) was applied for 40 min to dry the wells and any liquid left on the SPE cartridge tips was removed. The bound fraction was eluted by adding 900 μl of elution solvent (2% formic acid in methanol), followed by evaporation of the eluent at 70°C under nitrogen gas. The dried samples were reconstituted in 800 μl of mobile phase; acetonitrile-ammonium formate 20 mM with 1% formic acid (15/85, v/v).

Plasma (100 μl) was aliquoted into a 96-wellplate and diluted with 350 μl ammonium hydroxide (0.5 M) containing stable isotope-labeled internal standards (48.1 ng/ml of desethylchloroquine-D4 and 22.7 ng/ml of chloroquine-D4). The plate was mixed on a Mixmate at 1000 r.p.m. for 2 min and centrifuged at 1100 × *g* for 2 min (i.e., extraction-ready samples). The extraction samples (200 μl) were transferred to a supported liquid extraction, SLE^+^, 96-well plate. Vacuum of 3–4 inch Hg was applied for 30 s to allow the sample to absorb to the cartridge. The bound fraction was eluted with ethyl acetate (800 μl) followed by evaporation of the eluent at 70°C under nitrogen gas. The dried samples were reconstituted in 800 μl of mobile phase; acetonitrile-ammonium formate 20 mM with 1% formic acid (15–85, v/v).

From one DBS of approximately 50 μl, five discs of 3.2 mm in diameter were punched out (equivalent to 15 μl of whole blood) into a 96-wellplate. Acetonitrile-water with 0.5% formic acid (50-50, v/v; 200 μl) containing stable isotope-labeled internal standards (3.4 ng/ml of desethylchloroquine-D4 and 9.6 ng/ml of chloroquine-D4) was added to each sample, and the plate was mixed on a Mixmate at 1000 r.p.m. for 10 min and centrifuged at 1100 × *g* for 2 min. Acetonitrile (200 μl) was added to each sample and the plate was mixed on a Mixmate at 1000 r.p.m. for 2 min and centrifuged at 1100 × *g* for 2 min (i.e., extraction-ready samples). The extraction samples (250 μl) were loaded on a Phree Phospholipids Removal 96-wellplate. Vacuum was applied until the entire sample volume passed through the column, and the collected eluate was diluted with 170 μl of water.

### Instrumentation & chromatographic conditions

The LC system was an Agilent 1260 infinity system consisting of a binary LC pump, a vacuum degasser, a temperature-controlled microwell plate autosampler set at 4°C and a temperature-controlled column compartment set at 40°C (Agilent technologies, CA, USA). Data acquisition and processing were performed using Analyst 1.6.2 (Sciex, MA, USA). The analytes were separated on a Zorbax SB-CN 50 mm × 4.6 mm, I.D. 3.5 μm (Agilent Technologies), with a precolumn CN AJO-4305 4 mm × 3 mm, I.D. 3.5 μm (Phenomenex), at a flow rate of 700 μl/min. The mobile phase consisted of (A) acetonitrile-ammonium formate 20 mM with 1% formic acid pH approximately 2.6 (15–85, v/v) and (B) methanol–acetonitrile (75–25, v/v). The mobile phase gradient was A: 0–2 min, B: 2.2–3.7 min and A: 3.9–6.5 min (with 0.2 min linear gradient switch), resulting in a total runtime of 6.5 min per sample. The injection volume was 2 μl.

An API 5000 triple quadrupole mass spectrometer (Sciex) with a TurboV ionization source interface, operating in positive ion mode, was used for the MS/MS analysis. Ion spray voltage was set to 5500 V, with a drying temperature at 650°C. The curtain gas was 25 psi and the nebulizer (GS1) and auxiliary (GS2) gases were 60 psi.

### Validation procedure

The assays were validated according to the FDA, 2001 [32] and European Medicines Agency, 2012 on bioanalytical method validation [33].

Accuracy and precision of the methods were determined by analyzing five replicates of samples at the LLOQ and ULOQ, as well as quality control (QC) samples at three concentrations. Four (whole blood and plasma) to six (DBS) independent runs were performed and evaluated. Accuracy was calculated as mean relative error (%) by comparing the measured average concentration at each QC level with the nominal concentration. Precision of the method (within-run, between-run and total-assay variability) was calculated using a single factor analysis of variance (ANOVA), and expressed as the coefficient of variation (%). The ability to dilute samples above the ULOQ (i.e., dilution integrity of over the curve samples) was investigated by analyzing five replicates at 2–3 × ULOQ for chloroquine and desethylchloroquine by 1:5 dilutions for whole blood and DBS methods, and 1:10 dilutions for plasma method.

The calibration curve was assessed by analyzing four to six separate runs (the same as accuracy and precision determination). The best performing linear regression model (nonweighted, 1/x-weighted and 1/x^2^-weighted) was chosen based on the accuracy and precision of back-calculated concentrations of calibration standards and QC samples. Calibration standards and QC samples contributed equally to the selection of regression model by a ranking approach as previously described [36].

Selectivity was evaluated by analyzing six blank samples from six different donors for each matrix and the chromatograms were evaluated for any signal that potentially could interfere with the drug identification and measurement. Potentially interfering co-administered antimalarial drugs were investigated in a similar way by injecting 2 μl of individual piperaquine, pyronaridine, artesunate, primaquine and carboxyprimaquine at 30 ng/ml. The same experiment was then repeated while performing postcolumn infusion of chloroquine, desethylchloroquine and their stable isotope-labeled internal standards mix solution (20 ng/ml) for any signs of signal enhancement or suppression.

Absolute extraction recovery was determined by comparing the average response of extracted QC samples (five replicates at each level) with that of postextraction spiked blank blood samples at the same nominal concentration as the QC samples.

Matrix effects were investigated for different donors and anticoagulants using postcolumn infusion experiments. Blood from six different donors were collected using EDTA and from one of the donors, different anticoagulants (Na-heparin, Li-heparin, fluoride-heparin, citrate phosphate dextrose and fluoride oxalate) were also collected. All blank blood extracted samples from six different donors and different anticoagulants were investigated for ion suppression or enhancement caused by the matrix.

Matrix effects were also quantitatively determined by both matrix factor and normalized matrix factor and are described by Equations 1 and 2, respectively. Matrix factor is the ratio of analyte peak response of an extracted blank matrix sample spiked with analyte after extraction (*P_post-spiked_*) to the average analyte peak response of a reference solution at the same nominal concentration (*P_neat solution_*) [32 33]. Matrix factor for the internal standard is determined in the same way.

(Equation 1) $$Matrix\;factor=\frac{p_{post-spiked}}{p_{neat\;solution}}$$

The normalized matrix factor can be described as the ratio of matrix factor associated with the analyte to the matrix factor associated with the internal standard [32 33].

(Equation 2) $$Normalised\;matrix\;factor\;=\;\frac{Matrix\;factor_{analyte}}{Matrix\;factor_{internal\;standard}}$$

The calculated and normalized matrix factors below 0.85 (ion suppression) or above 1.15 (ion enhancement) would imply that a matrix effect was present.

Carryover effects were investigated by injecting five samples with drug concentrations at ULOQ followed by three blank mobile phase samples. A signal >20% the LLOQ in the injected blank samples would indicate carryover.

Stability of chloroquine and desethylchloroquine in whole blood, plasma and DBS was investigated by exposing the samples to five freeze and thaw cycles. The samples were frozen at -80°C for 24 h for the first freeze cycle and 12–24 h for the following freezing cycles and thawed at room temperature for 2–3 h. Short-term stability at room temperature (22°C) and at fridge temperature (4°C) was investigated at 4, 24 and 48 h. Long-term stability of spiked samples in storage condition (-80°C) was evaluated for at least 1 year. The stability of the analytes during the extraction process was evaluated for the following parameters; stability of extracted samples in extraction solution at 4°C for 24 h, stability of evaporated samples at 4°C for up to 72 h, and the stability in injection-ready samples in the LC autosampler at 4°C for up to 72 h. Spiked blood applied on Whatman 31 ET Chr paper was used to test different drying conditions of DBS in very high humidity conditions 27–32°C (88–92% relative humidity) and at dry conditions in a dry cabinet 20°C, 20% relative humidity. After spotting, the blood spots were left to dry for 1–2 h at ambient temperature and then transferred to different storage conditions. In wet tropical conditions, for example, rainy season, drying at ambient conditions is not possible and to simulate these conditions, the wet spot was directly transferred after blood spotting to a plastic bag and a desiccant was added to aid the drying process and the bag was then stored at ambient temperature. The DBS samples were left for 1–2 weeks in different environments and then analyzed against frozen (-80°C) reference samples.

The impact of using alternative filter papers with similar properties as Whatman 31 ET Chr was evaluated by spotting spiked blood on Whatman (DMPK-C, 903 Protein saver and 3 MM Chr) and Ahlstrom 226, and then compared them against Whatman 31 ET Chr as reference.

The impact of hematocrit was evaluated using the Whatman 31 ET Chr paper for DBS. After blood centrifugation (2000 g, 10 min), plasma was added or removed to achieve erythrocyte volume fraction of 20, 40 and 60%. Blood was then spiked and spotted (50 μl) and was allowed to dry completely before storage at -80°C with desiccant bag [37,38].

### Clinical applicability

All three validated methods were used in clinical studies to quantify chloroquine and desethylchloroquine drug concentrations.

## Results & discussion

### Method validation

#### Chromatographic separation & MS/MS optimization

The developed LC method allowed for complete separation of chloroquine and desethylchloroquine, with a total run time of 6.5 min, including the washout gradient. A relatively slow washout gradient was used to flush out any strongly retained compounds that might otherwise accumulate on the column and reduce the column performance over time, or co-elute with the analytes potentially causing matrix effects [39,40]. Identical LC methods were used for the analysis of samples from all three sample matrices (i.e., plasma, whole blood and DBS), enabling a simple and homogeneous method set up irrespective of sample matrix to be analyzed. Previously published bioanalytical methods (Table 1) show lower sensitivity and most often require a large sample volume to achieve adequate sensitivity, and they all have a longer analysis run time for each sample injection [19–25,28–31]. The simple separation method developed here results in excellent separation, using cleaner sample extraction techniques and shorter run time of 6.5 min for analysis and better sensitivity. All these advantages make this method ideal for implementation in routine drug analysis of large clinical pharmacokinetic trials.

**Table 1.** **Published assays of chloroquine and its active metabolite desethylchloroquine.**

**Drug/metabolite**	**Matrix**	**Sample volume (μl)**	**Extraction method**	**Detection method**	**Recovery (%)**	**Analysis run time**	**LLOQ**	**Ref.**
Chloroquine/desethylchloroquine	DBS	100	LLE	UV-Vis	72–92/101–105	12 min^†^	100/100 nmol/lL	[41]

Chloroquine/desethylchloroquine	DBS/whole blood	80/150	LLE	UV-Vis	DBS 75–78/75–76; whole blood 80–85/75–85	10 min^†^	DBS 50/50 ng/ml; whole blood 25/25 ng/ml	[28]

Chloroquine/desethylchloroquine	DBS	100	SPE	UV	72–82/75–78	40 min^†^	100/100 nmol/l	[42]

Chloroquine/desethylchloroquine	Plasma	500	LLE	DAD	83.7/92.3	14 min^†^	20/20 nmol/l	[43]

Chloroquine/desethylchloroquine	Plasma/whole blood	1000	SPE	DAD	Plasma 90–91/84–91; whole blood 78–82/99–102	14 min^†^	10/10 ng/ml	[29]

Chloroquine	Plasma	200	Protein precipitation	MS/MS	98–100	21 min	1.25 ng/ml	[30]

Chloroquine	DBS	10^‡^	Protein precipitation	MS/MS	115–122	19 min	20 ng/ml	[44]

Desethylchloroquine	Whole blood	100	Protein precipitation	MS/MS	87–90	9.5 min	25 ng/ml	[45]

^†^Approximate total runtime based on figures and chromatographic conditions.

^‡^Extraction of a 3 mm punched disc from a 10 μl DBS spot.

DAD: Diode array detector; DBS: Dry blood spot; LLE: Liquid–liquid extraction.

The MS/MS fragments were selected based on the most abundant transition signals (Supplementary Figures 1–4), compound purity, selectivity, sensitivity (as measured by signal-to-noise ratio) and analyte contribution. Chloroquine SRM transitions of m/z 320.2 > 247.2 contributed to chloroquine-D4 transition m/z 324.3 > 251.2 and resulted in inaccurate quantification data due to variations in the generated chloroquine-D4 signal. This phenomenon might occur if the isotope distribution of one compound overlay and interfere with another compound. In this case it is possible that one of the isotopes of chloroquine have a small contribution to chloroquine-D4 that will show its effect at high concentrations [46–49]. To prevent this mass interference, the second highest abundance transition m/z 324.3 > 146.3 was selected for chloroquine-D4. This transition had no interference with the chloroquine transition. Quantification was performed using SRM transitions of m/z 320.2 > 247.2 and 324.3 > 146.3 for chloroquine and chloroquine-D4, respectively, and 292.2 > 179.1 and 296.15 > 118.15 for desethylchloroquine and desethylchloroquine-D4, respectively. The collision energy was set to 29 V for all compounds. The developed detection method resulted in an unbiased robust method with high sensitivity. In some cases, a quantifier and a qualifier transition could increase the reliability of the results acquired. However, in this method, the reverse phase column has good retention and separation of chloroquine and desethylchloroquine, and this method also includes a sample clean-up step that further reduces the chance of interference. The quantifier–qualifier transition would be required if only protein precipitation and direct injection were used, with none or very little chloroquine and desethylchloroquine separation and retention. Thus, a qualifier transition would be more beneficial to avoid false result. The quantifier and qualifier transitions should also be fairly similar in signal, and for chloroquine, the SRM signal of qualifier transition was roughly a thirdof quantifier signal. This would work for most of the calibration range except around LLOQ level where the signal would be close to undetectable.

#### Recovery

The three different extraction techniques utilized for whole blood, DBS and plasma were aimed for high-throughput extraction and ease of use. The absolute recovery (extracted/postspiked) was in the range of 93–102% for whole blood, 56–64% for DBS and 69–80% for plasma at all QC levels tested for chloroquine and desethylchloroquine (Table 2). The absolute recovery of stable isotope-labeled internal standards was not affected by chloroquine and desethylchloroquine concentrations and was 103–109% for whole blood, 63–71% for DBS and 72–92% for plasma (Supplementary Table 1). The recoveries differed between matrices and the extraction technique utilized. For DBS recovery, cellulose fibres in Whatman 31 ET Chr (β-anhydroglucose units with dominant hydroxyl groups) could possibly interact with chloroquine and desethylchloroquine and hence affect the extraction efficiency in the DBS method, resulting in lower recovery than whole blood and plasma methods [50]. Moreover, three extraction techniques, SPE, SLE^+^ and phospholipid removal were evaluated for DBS samples, of which, phospholipid removal was the simplest method to use, which also gave the best recovery. For whole blood, only SPE using a CBA column gave clean enough extracts, which also produced the highest recoveries. For plasma, both phospholipid removal and SLE^+^ technique give similar recoveries; however, the SLE^+^ gave less phospholipid residues and the evaporated sample could also be dissolved in a mobile phase-optimized solution improving the chromatographic performance.

**Table 2.** **Absolute recovery, process efficiency and matrix effect of chloroquine and desethylchloroquine in human EDTA whole blood, plasma and dry blood spot sample.**

**Matrices**	**Drug**	**Concentration (ng/ml)**	**Absolute recovery (%)**	**Process efficiency (%)**	**CV (%)**	**Matrix factor**	**Normalized matrix factor (drug/IS)**	**CV (%)**
Whole blood	Chloroquine	QC 1 (7.56)	102	97.3	1.54	0.959	1.01	1.50

		QC 3 (1049)	95.7	86.2	3.52	0.901	0.985	2.85

	Desethylchloroquine	QC 1 (9.40)	98.5	92.5	3.17	0.939	0.991	4.06

		QC 3 (1049)	93.5	86.3	2.95	0.923	1.02	1.81

DBS	Chloroquine	QC 1 (6.03)	56.2	59.1	7.87	1.05	1.00	4.06

		QC 3 (1334)	56.6	59.3	6.76	1.05	1.00	2.18

	Desethylchloroquine	QC 1 (8.89)	64.4	69.2	5.58	1.07	1.04	6.06

		QC 3 (1334)	62.5	64.5	6.38	1.03	0.990	2.04

Plasma	Chloroquine	QC 1 (4.64)	72.9	75.5	8.01	1.04	1.03	5.29

		QC 3 (524)	68.7	68.8	9.10	1.00	0.990	3.87

	Desethylchloroquine	QC 1 (4.64)	79.6	77.7	5.51	0.977	0.967	5.59

		QC 3 (524)	76.2	78.4	1.76	1.03	1.03	4.12

DBS: Dried blood spot; IS: Internal standard; QC: Quality control.

Many of the previously published methods using UV and fluorescence detector show good recovery, but in most cases very basic sample preparation techniques were applied (e.g., protein precipitation and liquid–liquid extraction), and residual proteins and phospholipids can often lead to reduced LC column efficiency and LC–MS/MS matrix effects. Also a large sample volume for analysis requires more blood to be collected, which is a drawback if studies also involve young children [17,20–25,27]. Moreover, longer runtime for analysis makes these methods not suitable for high-throughput analysis of large sample batches from pharmacokinetic studies [21,23,28,29].

#### Selectivity & matrix effects

**Figure 2.**
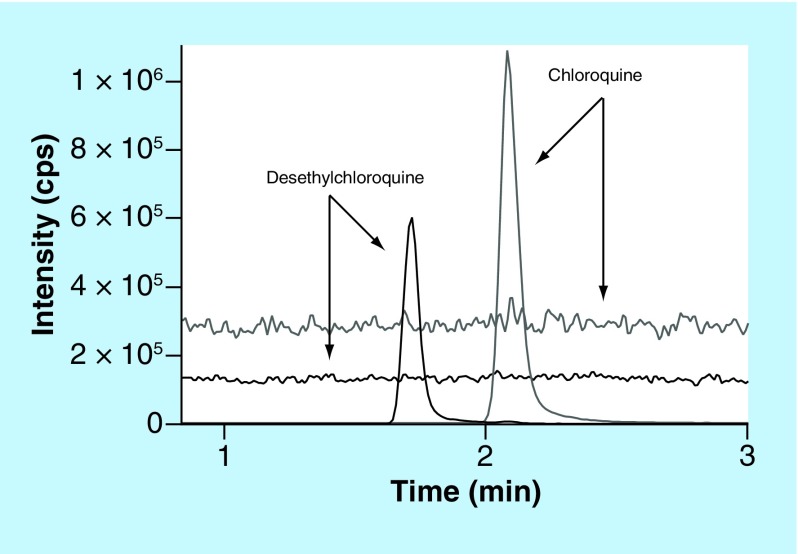
**Overlay of ULOQ concentrations of chloroquine and desethylchloroquine and blank EDTA dried blood spot extracted sample injected during postcolumn infusion 10 μl/min with a mixed solution of chloroquine and desethylchloroquine (20 ng/ml).**

None of the blank sources of sample matrices produced a signal that contributed >20% compared with that of a standard sample at LLOQ, demonstrating a high selectivity of the developed methods with a minimal risk of interference from different patient matrices. All blank sources were also free from signals generating ion suppression/enhancement of analytes or internal standards. Postcolumn infusion did not show any signs of ion suppression/enhancement for chloroquine or desethylchloroquine and their stable isotope-labeled internal standards (Figure 2, represents a DBS-extracted sample injected during postcolumn infusion), generating a matrix factor close to one for all compounds. Minor ion suppression/enhancement was fully compensated by the stable isotope-labeled internal standards, resulting in normalized matrix effects close to one (with low variation) for both chloroquine and desethylchloroquine (Table 2). Injecting commonly used antimalarial drugs (potentially co-administered in a clinical setting) in a normal LC run did not produce any interfering peaks (data not shown) and should not have any impact on the quantification of chloroquine and desethylchloroquine. The second metabolite of chloroquine, bisdesethylchloroquine, was not included in this method as it is found in much lower concentrations and not widely used in pharmacokinetic studies. However, although we do not know the retention time of bisdesethylchloroquine, the chance of it interfering would be considered very low, as it would very likely have separated on the column. The difference of chloroquine and desethylchloroquine (which both are well separated) is an ethyl group; hence, a loss of another ethyl group as in bisdesethylchloroquine would change the retention even further. Moreover, since bisdesethylchloroquine has a different mass; it would be separated in the mass spectrometer as well.

The additional method evaluation tests performed for DBS demonstrated that it is crucial that the blood spot sample to have soaked through the paper completely and that the blood spot is homogeneous and of appropriate size to ensure that the same volume of blood is generated with each punch. Filter papers with similar properties to the Whatman 31 ET Chr can be used as alternative (i.e., Whatman DMPK-C, 903 Protein as well as Ahlstrom 226). The thinner and denser Whatman 3 MM Chr paper may cause blood to flow out as a noncircular and nonhomogeneous spot, especially at high hematocrit, and is therefore not recommended for the DBS quantification method. The thin paper also absorbed less blood per surface area, causing a blood volume bias when blood discs were punched out from the blood spot compared with the thicker papers. Obviously, mixing papers with different properties in a clinical trial setting will affect the quantification, resulting in incorrect concentrations and loss of accuracy and precision. The different hematocrit levels evaluated (20, 40 and 60%) might affect the physical size of the blood spot depending on the paper used and hence the amount of blood obtained in a punched disc. DBS methods for antimalarial drug studies should be able to cover studies from healthy volunteers to clinical malaria cases with anemia, and the hematocrit range will therefore be very wide. Malaria patients usually have low hematocrit and in severe malaria hematocrit below 20% can be found [51]. However, using whole blood at different hematocrit levels to create DBS and comparing it to a calibration curve with a hematocrit of 45% did not show any major impact on the quantification of chloroquine or desethylchloroquine. All DBS samples were within ±15% deviation limit and met the acceptance criteria (Table 3 & Supplementary Table 2).

**Table 3.** **Impact of hematocrit level for chloroquine and desethylchloroquine in human EDTA dried blood spot (n = 4).**

**Analyte**	**Concentration (ng/ml)**	**Hematocrit (%)**	**Average concentration (ng/ml)**	**Accuracy (%)**	**Precision (% CV)**
Chloroquine	6.03	20	5.97	98.9	14.8

		40	5.73	95.0	6.57

		60	6.28	104	2.83

	1334	20	1165	87.3	2.67

		40	1318	98.8	2.73

		60	1345	101	3.46

Desethylchloroquine	8.89	20	8.74	98.3	10.3

		40	8.37	94.1	9.43

		60	8.46	95.2	5.31

	1334	20	1120	84.0	2.63

		40	1235	92.6	4.88

		60	1275	95.6	1.87

Furthermore, punching in center or close to the edge of the DBS did not have any impact on the quantification of chloroquine or desethylchloroquine. Overall, the developed DBS method showed robust performance over a range of different sampling papers, hematocrit levels and location of the paper punch (Supplementary Table 2).

#### Precision & accuracy

**Figure 3.**
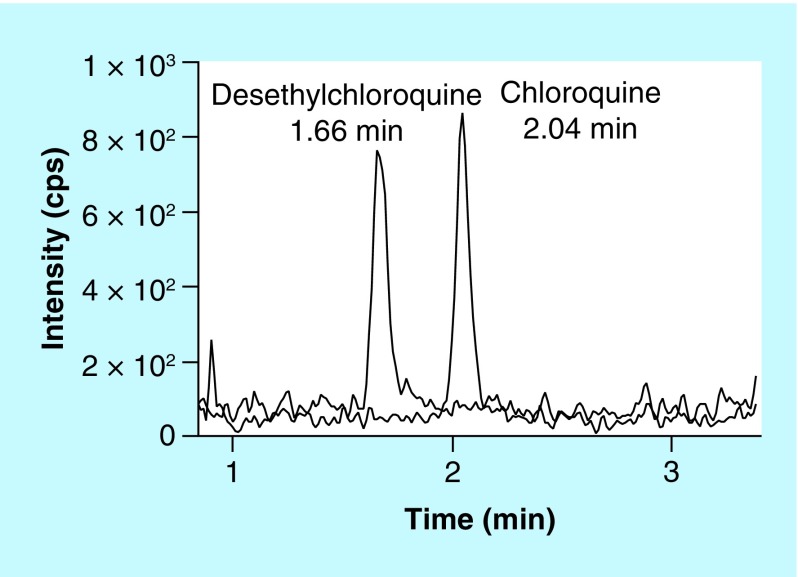
**Extracted ion chromatogram of an analysed dried blood spot sample containing LLOQ concentrations of chloroquine (1.82 ng/ml) and desethylchloroquine (2.95 ng/ml), overlaid with a blank sample.**

The ULOQ values were chosen based on available pharmacokinetic data to cover the range of maximum concentrations reported in patients after receiving approximately total dose of 25 mg/kg of chloroquine base but also avoiding carryover and mass detector saturation [28,52–54]. Chloroquine and desethylchloroquine accumulate in erythrocyte cells giving higher concentrations in whole blood compared with plasma [17,18,55]. Therefore, the concentration range and ULOQ values were lower in the plasma method compared with the whole blood or DBS methods. No carryover was detected for the developed methods. The selected LLOQ values were clearly visible and gave a response of at least five-times compared with the blank response and easily detected by all three methods [32]. This is illustrated by a representative chromatogram of a DBS sample containing 1.82 ng/ml of chloroquine and 2.95 ng/ml of desethylchloroquine (Figure 3), resulting in greater than or equal to tenfold signal-to-noise response.

Ordinary linear regression with 1/x^2^ weighting resulted in the best prediction of calibration standards and QC samples, and a high correlation coefficient (r >0.99). Accuracy and precision of the methods are presented in Table 4. Both accuracy and precision were well within the allowed regulatory criteria of <15% deviation.

**Table 4.** **Accuracy and precision for chloroquine and desethylchloroquine extracted from human EDTA whole blood, plasma and dried blood sample.**

**Sample type**	**Chloroquine**					**Desethylchloroquine**				

	**Nominal concentration (ng/ml)**	**Measured concentration (ng/ml)**	**Accuracy (%)**	**Precision (%)**		**Nominal concentration (ng/ml)**	**Measured concentration (ng/ml)**	**Accuracy (%)**	**Precision (%)**	

				**Between run CV (interassay)**	**Within run CV (intra-assay)**				**Between run CV (interassay)**	**Within run CV (intra-assay)**
**Whole blood (n = 5, b = 4)**										

LLOQ	2.56	2.63	103	7.27	5.13	3.36	3.25	96.9	6.97	4.14

QC1	7.56	7.70	102	3.46	2.74	9.40	9.70	103	3.92	2.57

QC2	91.5	94.9	104	2.11	2.78	102	106	104	2.79	1.60

QC3	1049	1093	104	2.49	1.92	1049	1090	110	2.78	1.58

ULOQ	1220	1233	101	3.40	3.59	1220	1244	102	2.49	3.76

Over curve^†^	2556	2681	104	2.52	2.77	2556	2650	104	5.68	2.62

**DBS (n = 5, b = 6)**										

LLOQ	1.82	1.95	107	16.8	14.0	2.95	3.21	109	15.7	12.4

QC1	6.03	5.76	95.5	5.20	7.09	8.89	8.40	94.5	14.2	7.79

QC2	102	97.9	96.0	5.90	4.29	124	112	90.2	7.97	4.48

QC3	1334	1323	99.2	4.56	5.21	1334	1285	96.4	7.65	3.62

ULOQ	1552	1590	102	4.29	4.54	1552	1566	101	3.11	3.26

Over curve	3429	3511	102	6.97	3.41	3429	3453	101	6.30	5.25

**Plasma (n = 5, b = 4)**										

LLOQ	1.41	1.45	103	10.1	9.64	1.41	1.43	101	6.96	12.6

QC1	4.64	4.35	93.7	5.65	6.14	4.64	4.34	93.5	3.40	5.21

QC2	56.4	55.3	98.1	5.94	3.71	56.4	53.0	94.0	3.88	3.98

QC3	524	509	97.2	6.51	3.28	524	493	94.1	4.78	4.22

ULOQ	610	597	97.9	7.53	4.02	610	598	98.0	1.31	3.51

Over curve	1932	1953	101	5.00	2.87	1932	1939	100	5.12	4.13

Over curve, that is, sample dilution integrity test.

^†^Over-curve whole blood sample diluted five-times with blank plasma.

b: Number of runs; DBS: Dried blood spot; n: Number of samples in each run; QC: Quality control.

#### Stability

Chloroquine and desethylchloroquine in whole blood and plasma, were stable at ambient temperature for 4 h, as well as at 4°C for at least 48 h. Both analytes were stable for five freeze/thaw cycles in whole blood, DBS and plasma. Postextraction reconstituted samples at 4°C (i.e., samples ready for injection in the autosampler) were stable for at least 34 h in whole blood method, 48 h in DBS method and 74 h in plasma method. Chloroquine and desethylchloroquine stock solutions (neat solution) were stable for at least 6 h at ambient temperature, at least 2 weeks at 4°C, and at least 1 year in long-term storage (-80°C). The long-term storage stability (-80°C) of chloroquine/desethylchloroquine in whole blood and plasma was at least 3.1 and 1.2 years, respectively. Chloroquine and desethylchloroquine in DBS were stable at ambient temperature (below 20% relative humidity, stored with desiccant) for at least 1.2 years. The duration of evaluated long-term stability was dependent on actual storage of spiked samples, and it does not mean that chloroquine and desethylchloroquine is more stable in whole blood compared with plasma at similar storage conditions.

Different drying conditions were evaluated for DBS samples (i.e., fast/slow drying [1–6 h] in low/high humidity [40–80% relative humidity]), and demonstrated no major impact on stability and subsequent quantification (Supplementary Table 3). Humid conditions should be avoided to prevent fungus growth, which might affect drug quantification. However, DBS samples should dry completely before long-term storage in plastic bags in the presence of a desiccant. Samples can then be stored at room temperature as long as they are kept dry. The use of a desiccant is required to ensure sample integrity when samples are collected at tropical field sites under very humid conditions.

Overall, chloroquine and desethylchloroquine showed high stability in all of the three biological matrices evaluated (whole blood, DBS and plasma), with the possibility of long-term storage of clinical samples for at least 1–3 years. No part of the developed methods was particularly sensitive to stability issues, and it should be straight forward to implement these methods in routine sample analysis.

#### Clinical applicability of the developed & validated methods

**Figure 4.**
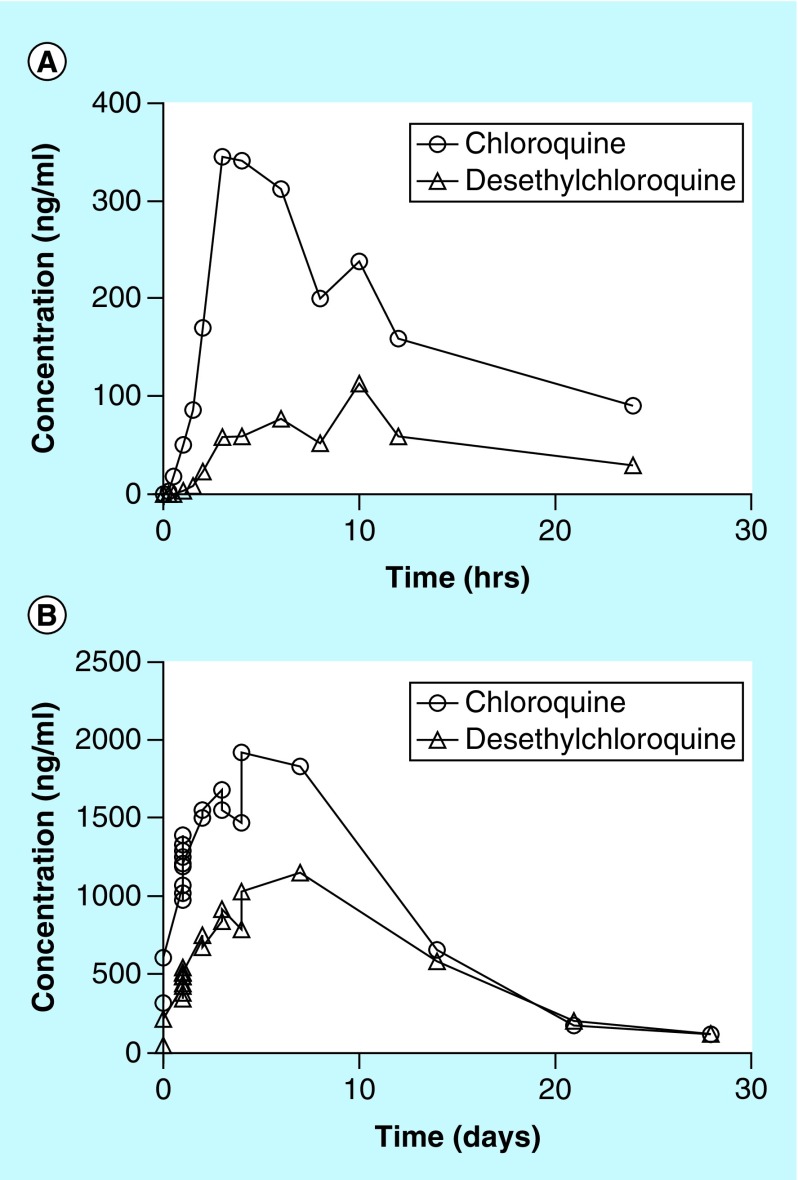
**Pharmacokinetic concentration-time profile of chloroquine and desethylchloroquine in plasma and DBS.** **(A)** plasma from a healthy Thai volunteer receiving an oral single dose of chloroquine (600 mg), and **(B)** dried blood spot from a study of a young child (22 kg) with vivax malaria after treatment with a high oral chloroquine dose (1550 mg) over 5 days.

The developed methods were easily implemented into high-throughput routine sample analysis setting, using a liquid handler platform and three parallel LC–MS/MS machines. All methods proved reliable and robust performance for the analysis of chloroquine and desethylchloroquine in whole blood, DBS and plasma patient samples from clinical trials. The whole blood method was used to analyze 1600 samples from a vivax malaria recurrence study. The DBS method was used to analyze 620 samples from children treated with chloroquine for vivax malaria and the plasma method was used to analyze 182 plasma samples from a healthy volunteer study. The selected calibration range for plasma, whole blood and DBS proved suitable for the concentration measurements of the study samples. Below is a representative graph of a pharmacokinetic concentration-time profile in plasma samples from an adult (Figure 4A) receiving a single oral dose of chloroquine (600 mg) and from a separate study, a child (Figure 4B) bodyweight 22 kg receiving oral chloroquine with a total dose of 1550 mg over 5 days and samples collected as DBS, analyzed using the developed methods. Chloroquine and desethylchloroquine gave maximum concentrations similar to previously described studies [52–54,56], which also reported a C_max_ after 3–9 h and a slow elimination phase due to high volume of distribution into the body's adipose tissue giving a long terminal elimination half-life [16,52,53,57]. Chloroquine concentrations were higher than its metabolite desethylchloroquine (Figure 4A & B); however, both have the same activity against parasites [58]. Chloroquine is giving higher concentrations in DBS compared with plasma due to its ability to accumulate in red blood cell [16–18]. The reliability of all validated methods was confirmed by repeated analysis of some of the patient samples, known as incurred sample reanalysis [59]. For incurred sample reanalysis evaluation, patient samples were selected across the concentration profile and analyzed in a separate run [59]. Furthermore, the DBS method enabled capillary finger-prick sampling in a pediatric field study, a pharmacokinetic study that would not have been practically or ethically possible without the development of this analysis method.

## Conclusion

The high selectivity and sensitivity of the developed methods, combined with a short run time and small sample volume, are a substantial improvement compared with previously published methods. The developed methods use different sample extraction techniques to simplify the extraction procedure for each matrix. However, all methods use identical separation and detection techniques to maximize the sample throughput and allow for implementation of methods in a high-throughput routine clinical trial setting. The DBS methodology enables clinical field trials in vulnerable populations where large volumes are not ethically and/or practically possible, such as small children. Since no centrifugation or cold chain is needed, it also enables pharmacokinetic studies to be performed in rural field sites and at a lower cost. All methods were demonstrated to be robust and reliable when used for drug quantification in clinical patient samples from large pharmacokinetic clinical trials.

## Future perspective

Highly sensitive and selective bioanalytical quantification methods are essential when analysing clinical sample particularly where larger sample volumes are not ethical or practically possible, such as small children. These bioanalytical methods using LC–MS/MS presented here, provided rugged and robust results in both method validation and clinical samples. These new methods are easy to automate, and consequently will reduce the time for processing large number of clinical pharmacokinetic samples. The sample extraction and clean-up step incorporates modern faster sample processing techniques such as, phospholipids removal 96-well plate for DBS sample, and supported liquid extraction 96-well plate for plasma sample. Moreover, clinical studies for malaria treatment are often located in rural areas where sample storage and transportation are more challenging making DBS as a sample collection technique more important. The DBS technique will not only save time and money, it is also very suitable for vulnerable populations such as small children with only a small amount of blood required for analysis.

**Summary points**

**Background**

- A new set of bioanalytical methods, using LC–MS/MS, to quantify chloroquine and its metabolite, desethylchloroquine, in various human blood samples was developed and validated.

**Experimental**

- High-throughput extraction techniques were used to recover chloroquine and its metabolite, desethylchloroquine, from whole blood (50 μl), plasma (100 μl) and whole blood (50 μl) applied on filter paper as dried blood spot (DBS) sample (a DBS sample was punched out, equivalent to 15 μl of whole blood) followed by quantification with LC–MS/MS.
- The three high-throughput extraction techniques were selected for determination of chloroquine and desethylchloroquine, and consist of two new high-throughput techniques, phospholipids removal 96-well plate for DBS sample, supported liquid extraction 96-well plate for plasma sample, and a more selective technique of sample extraction technique 96-well plate with carboxylic acid bonded sorbent (CBA) as an ion-exchange sorbent for whole blood sample.
- The LC method was the same for all three methods and used a reverse phase column achieving full baseline separation, which resulted in a total runtime of 6.5 min per sample including the wash out gradient.

**Results & discussion**

- All bioanalytical methods were robust with high sensitivity and selectivity. The intrabatch and interbatch precisions were below 15% (below 20% for the LLOQ) for all compounds and sample matrices, and thus meet regulatory acceptance criteria.
- LLOQ of chloroquine/desethylchloroquine was 2.56/3.36 ng/ml, 1.41/1.41 ng/ml and 1.82/2.95 ng/ml in whole blood, plasma and DBS respectively.

**Conclusion**

- All methods demonstrated satisfactory validation performance with high sensitivity and selectivity, and using simple extraction techniques that are easy to automate. These methods are suitable for implementation into high-throughput routine analysis of clinical pharmacokinetic samples.
