# Supplementary material for: High sensitivity methods to quantify chloroquine and its metabolite in human blood samples using LC–MS/MS
Source: Bioanalysis. 2019 Mar 15;11(5):333–47. doi: 10.4155/bio-2018-0202 (PMC6562699; doi:10.4155/bio-2018-0202)
Supplement: Supplementary file 1 [file bio-11-333-s1.docx]

**Bioanalysis Journal**

**Electronic Supplementary Information to:**

**High sensitivity methods to quantify chloroquine and its metabolite in human blood samples using LC-MS/MS**

**Figure S1** Chloroquine: Collision energy scan and fragmentation of chloroquine (320 m/z).

**Figure S2** Stable isotope labelled internal standard chloroquine-D4: Collision energy scan and fragmentation of chloroquine-D4 (324 m/z).

**Figure S3** Desethylchloroquine: Collision energy scan and fragmentation of desethylchloroquine (292 m/z).

**Figure S4** Stable isotope labelled internal standard desethylchloroquine-D4: Collision energy scan and fragmentation of desethylchloroquine-D4 (296 m/z).

**Table S1** Absolute recovery, process efficiency and matrix effect of stable isotope-labelled internal standards; chloroquine-D4 and desethylchloroquine-D4 in human EDTA whole blood, plasma and DBS sample.

| **Matrices** | **Drug** | **Concentration (ng/mL)** | **Absolute recovery (%)** | **Process efficiency (%)** | **CV (%)** | **Matrix factor** |
| --- | --- | --- | --- | --- | --- | --- |
| Whole blood | Chloroquine-D4 | SIL QC 1 (72.45) | 104 | 99.3 | 2.83 | 0.954 |
|  |  | SIL QC 3 (72.45) | 109 | 99.8 | 3.00 | 0.915 |
|  | Desethylchloroquine-D4 | SIL QC 1 (25.8) | 103 | 97.7 | 2.42 | 0.947 |
|  |  | SIL QC 3 (25.8) | 106 | 96.6 | 2.36 | 0.908 |
| DBS | Chloroquine-D4 | SIL QC 1 (9.66) | 62.7 | 65.7 | 3.94 | 1.05 |
|  |  | SIL QC 3 (9.66) | 65.1 | 68.3 | 7.84 | 1.05 |
|  | Desethylchloroquine-D4 | SIL QC 1 (3.44) | 67.3 | 69.5 | 3.13 | 1.03 |
|  |  | SIL QC 3 (3.44) | 70.9 | 73.9 | 6.60 | 1.04 |
| Plasma | Chloroquine-D4 | SIL QC 1 (8.08) | 81.0 | 81.7 | 8.17 | 1.01 |
|  |  | SIL QC 3 (8.08) | 71.6 | 72.5 | 9.93 | 1.01 |
|  | Desethylchloroquine-D4 | SIL QC 1 (8.08) | 91.6 | 92.7 | 6.03 | 1.01 |
|  |  | SIL QC 3 (8.08) | 85.2 | 85.2 | 5.83 | 0.999 |

CV, coefficient of variation; DBS, dried blood spot; QC, quality control and SIL, stable isotope-labelled internal standards.

**Table S2** Average of spot punches (*n=4*) centre vs side punch and percentage of haematocrit level for chloroquine and desethylchloroquine in human EDTA dried blood spot.

| **Drug** | **Concentration** | **50 µL 20%HCT** | **50 µL 40%HCT** | **50 µL 60%HCT** |  | **100 µL centre punch 20%HCT** | **100 µL side punch 20%HCT** | **100 µL centre punch 40%HCT** | **100 µL side punch 40%HCT** | **100 µL centre punch 60%HCT** | **100 µL side punch 60%HCT** |
| --- | --- | --- | --- | --- | --- | --- | --- | --- | --- | --- | --- |
| **Chloroquine** | **6.03 ng/mL** | 5.97 | 5.73 | 6.28 |  | 5.53 | 5.25 | 6.21 | 5.55 | 6.74 | 6.45 |
|  | **CV (%)** | 14.8 | 6.57 | 2.83 |  | 8.28 | 9.00 | 10.9 | 4.44 | 8.81 | 7.95 |
|  | **Accuracy** | 98.9 | 95.0 | 104 |  | 91.7 | 87.0 | 103 | 92.0 | 112 | 107 |
|  | **1334 ng/mL** | 1165 | 1318 | 1345 |  | 1245 | 1223 | 1313 | 1310 | 1415 | 1420 |
|  | **CV (%)** | 2.67 | 2.73 | 3.46 |  | 3.11 | 3.30 | 3.07 | 3.58 | 1.87 | 3.54 |
|  | **Accuracy** | 87.3 | 98.8 | 101 |  | 93.3 | 91.6 | 98.4 | 98.2 | 106 | 106 |
| **Desethylchloroquine** | **8.89 ng/mL** | 8.74 | 8.37 | 8.46 |  | 8.07 | 7.16 | 8.58 | 8.37 | 9.03 | 8.65 |
|  | **CV (%)** | 10.3 | 9.43 | 5.31 |  | 8.17 | 6.26 | 4.30 | 9.78 | 9.26 | 4.89 |
|  | **Accuracy** | 98.3 | 94.1 | 95.2 |  | 90.7 | 80.5 | 96.5 | 94.1 | 102 | 97.3 |
|  | **1334 ng/mL** | 1120 | 1235 | 1275 |  | 1235 | 1150 | 1255 | 1273 | 1400 | 1253 |
|  | **CV (%)** | 2.63 | 4.88 | 1.87 |  | 5.06 | 5.55 | 1.66 | 2.43 | 4.74 | 2.20 |
|  | **Accuracy** | 84.0 | 92.6 | 95.6 |  | 92.6 | 86.2 | 94.1 | 95.4 | 105 | 93.9 |

Four replication of sample analysis

HCT, haematocrit and CV, coefficient of variation.

**Table S3** Average drying stability of chloroquine and desethylchloroquine in EDTA DBS sample.

| **Drug** | **Concentration** | **-80°C without desiccant** | **-20 °C without desiccant** | **- 20 °C +desiccant** | **RT + desiccant** | **14 Days Open air (>80%RH), 30°C** | **14 Days Dried in plastic bag + desiccant (>80%RH), 30°C** |
| --- | --- | --- | --- | --- | --- | --- | --- |
| **Chloroquine** | **6.03 (ng/mL)** | 5.85 | 5.67 | 5.69 | 6.14 | 5.42 | 5.37 |
|  | **CV (%)** | 10.6 | 6.17 | 10.4 | 15.9 | 3.35 | 7.61 |
|  | **Accuracy** | 97.0 | 94.1 | 94.4 | 102 | 89.9 | 89.1 |
|  | **1334 (ng/mL)** | 1230 | 1327 | 1267 | 1343 | 1270 | 1183 |
|  | **CV (%)** | 1.63 | 2.18 | 3.29 | 4.23 | 1.57 | 6.78 |
|  | **Accuracy** | 92.2 | 99.5 | 95.0 | 101 | 95.2 | 88.7 |
| **Desethylchloroquine** | **8.89 (ng/mL)** | 8.02 | 8.02 | 8.24 | 8.48 | 7.69 | 7.84 |
|  | **CV (%)** | 5.00 | 7.43 | 7.40 | 8.92 | 3.75 | 5.44 |
|  | **Accuracy** | 90.2 | 90.2 | 92.7 | 95.4 | 86.5 | 88.2 |
|  | **1334 (ng/mL)** | 1250 | 1263 | 1257 | 1293 | 1190 | 1167 |
|  | **CV (%)** | 1.39 | 1.99 | 6.95 | 0.893 | 4.20 | 4.87 |
|  | **Accuracy** | 93.7 | 94.7 | 94.2 | 97.0 | 89.2 | 87.5 |

Triplicate of sample analysis

RT, room temperature (25 °C); RH, relative humidity and CV, coefficient of variation.
